# Inhibition of Aberrant α(1,2)-Fucosylation at Ocular Surface Ameliorates Dry Eye Disease

**DOI:** 10.3390/ijms22157863

**Published:** 2021-07-23

**Authors:** Chang Ho Yoon, Jin Suk Ryu, Jung Hwa Ko, Joo Youn Oh

**Affiliations:** 1 Department of Ophthalmology, College of Medicine, Seoul National University, 103 Daehak-ro, Jongno-gu, Seoul 03080, Korea; ifree7@gmail.com; 2 Laboratory of Ocular Regenerative Medicine and Immunology, Biomedical Research Institute, Seoul National University Hospital, 101 Daehak-ro, Jongno-gu, Seoul 03080, Korea; enter2357@naver.com (J.S.R.); kjh382@hanmail.net (J.H.K.)

**Keywords:** 2-deoxy-D-galactose, dry eye disease, fucosylation, glycosylation, ocular surface

## Abstract

Fucosylation is involved in a wide range of biological processes from cellular adhesion to immune regulation. Although the upregulation of fucosylated glycans was reported in diseased corneas, its implication in ocular surface disorders remains largely unknown. In this study, we analyzed the expression of a fucosylated glycan on the ocular surface in two mouse models of dry eye disease (DED), the NOD.B10.H2^b^ mouse model and the environmental desiccating stress model. We furthermore investigated the effects of aberrant fucosylation inhibition on the ocular surface and DED. Results demonstrated that the level of type 2 H antigen, an α(1,2)-fucosylated glycan, was highly increased in the cornea and conjunctiva both in NOD.B10.H2^b^ mice and in BALB/c mice subjected to desiccating stress. Inhibition of α(1,2)-fucosylation by 2-deoxy-D-galactose (2-D-gal) reduced corneal epithelial defects and increased tear production in both DED models. Moreover, 2-D-gal treatment suppressed the levels of inflammatory cytokines in the ocular surface and the percentages of IFN-γ^+^CD4^+^ cells in draining lymph nodes, whereas it did not affect the number of conjunctival goblet cells, the MUC5AC level or the meibomian gland area. Together, the findings indicate that aberrant fucosylation underlies the pathogenesis of DED and may be a novel target for DED therapy.

## 1. Introduction

Fucosylation, a type of glycosylation, is one of the most common post-translational modifications involved in many basic cellular biological processes such as cell adhesion, recognition, development and host–microbe interactions [1,2]. In recent years, the important roles of fucosylation in immune cell development and function have been uncovered [1,3]. Evidence is mounting that aberrant fucosylation plays a critical role in a number of inflammatory conditions such as rheumatoid arthritis [4,5], chronic pancreatitis [6], Crohn’s disease [7,8], type I diabetes [9] and allergic airway inflammation [10], as well as in cancer [11,12,13].

At the ocular surface, glycosylation has long been considered an important factor in the regulation of homeostasis [14,15,16]. It is well known that mucin-type O-glycans and N-glycans are expressed in the tear film and the ocular surface epithelia [15,16,17,18,19]. It was also reported that fucosylated glycans were expressed in normal corneas and conjunctivas [20,21,22,23], and their expression was upregulated in diseased corneas [23]. Given a range of functions of fucosylated glycans in physiologic and pathologic cellular processes, it is conceivable that fucosylation might play a role in ocular surface health and disease. However, little is known about the role of fucosylation at the ocular surface.

In this study, we aimed to test whether altered terminal α(1,2)-fucosylation occurs at the ocular surface in dry eye disease (DED) and is involved in the disease pathogenesis. First, we analyzed the levels of type 2 H antigen (H2 antigen), an α(1,2)-fucosylated carbohydrate, in the cornea and conjunctiva in two murine DED models: the NOD.B10.H2^b^ mouse model and the environmental desiccating stress model. Next, we investigated the functional consequences of inhibition of α(1,2)-fucosylation by 2-deoxy-D-galactose (2-D-gal) on the ocular surface in DED.

## 2. Results

### 2.1. H2 Antigen Is Upregulated at Ocular Surface in DED Mice

**Figure 1:**
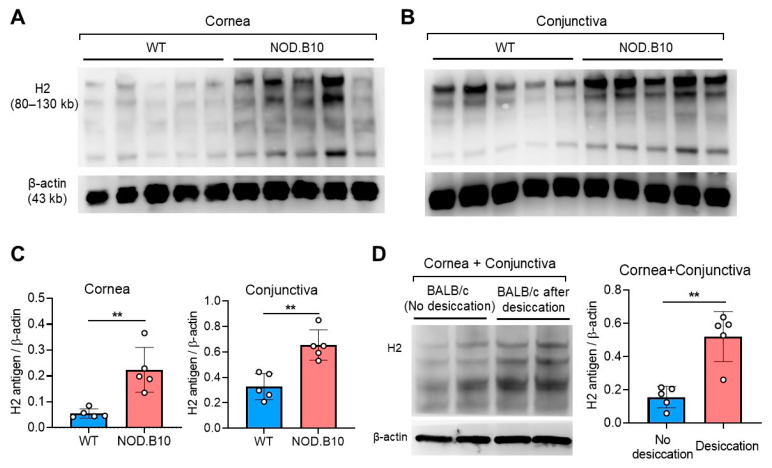
H2 antigen expression in the cornea and conjunctiva in two murine DED models. (**A**,**B**) Western blot images for H2 antigen in the cornea (**A**) and conjunctiva (**B**) in wild-type (WT) C57BL/6 and NOD.B10.H2^b^ (NOD.B10) mice at 12 weeks of age. (**C**) Densitometric analysis of the ratio of H2 antigen expression relative to β actin. (**D**) Representative Western blot image and quantitative densitometric analysis of H2 antigen in the cornea and conjunctiva in 30-week-old BALB/c mice under physiological condition (without desiccating stress) or under desiccating stress. A circle represents the data from a single individual animal. Bar depicts mean ± SD. ** *p* < 0.01.

We measured the levels of H2 antigen in the cornea and conjunctiva in two murine DED models and compared with those in control mice without DED (Figure 1). Western blot analysis demonstrated the expression of H2 antigen in the cornea and conjunctiva in wild-type (WT) C57BL/6 and BALB/c mice. Remarkably, the protein levels of H2 antigen in the cornea and conjunctiva were increased in NOD.B10.H2^b^ mice, compared to control C57BL/6 mice (Figure 1A–C). Similarly, H2 antigen levels were elevated in BALB/c mice after 2 weeks of desiccating stress, compared to BALB/c mice without desiccating stress (Figure 1D). These results suggest that aberrant upregulation of α(1,2)-fucosylation occurs at the ocular surface both in ocular Sjögren’s syndrome (SjS)-like DED and environmental desiccating stress-induced DED.

### 2.2. 2-D-Gal Alleviates Clinical Signs of DED

**Figure 2:**
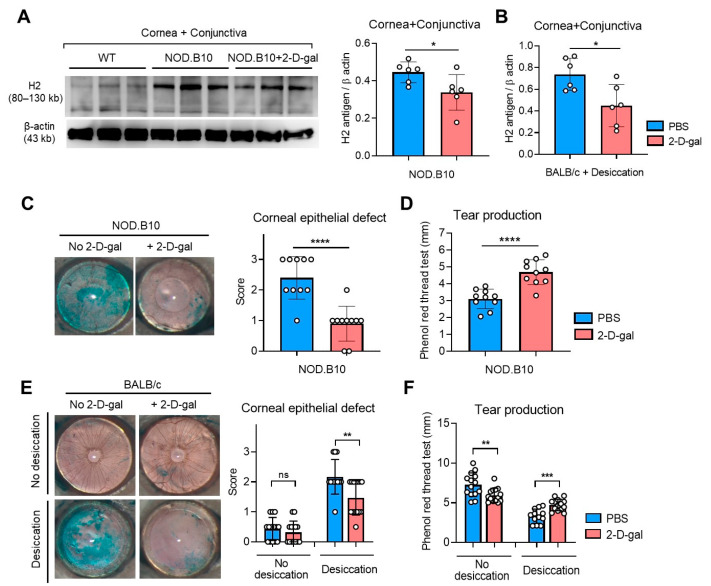
Therapeutic effects of α(1,2)-fucosylation blockade by 2-D-gal on clinical signs of DED in two mouse models. (**A**) Western blot images and densitometric analysis of H2 antigen relative to β actin in the cornea and conjunctiva in wild-type (WT) C57BL/6 mice and NOD.B10.H2^b^ (NOD.B10) mice with or without 2-D-gal treatment. (**B**) Densitometric results of Western blot analysis for H2 antigen relative to β actin in the cornea and conjunctiva in BALB/c mice under desiccating stress with or without 2-D-gal treatment. (**C**) Representative corneal photographs after Lissamine Green vital dye staining in NOD.B10 mice with or without 2-D-gal treatment. Quantification of corneal epithelial defects as graded by the standardized 4-point scoring system (0–3). (**D**) Tear production measurement by using a phenol red thread test in NOD.B10 mice with or without 2-D-gal treatment. (**E**) Representative corneal photographs and quantification of corneal epithelial defects in BALB/c mice under physiological (no desiccation) or desiccating stress condition with or without 2-D-gal treatment. (**F**) Assay for tear production by a phenol red thread test in BALB/c mice (under physiological or desiccating condition) with or without 2-D-gal treatment. A circle depicts data (mean ± SD) from an individual animal. * *p* < 0.05, ** *p* < 0.01, *** *p* < 0.001, **** *p* < 0.0001, ns: not significant.

Intraperitoneal (IP) administration of 2-D-gal significantly decreased H2 antigen levels in the cornea and conjunctiva (Figure 2A,B). Along with decreased H2 antigen, corneal epithelial defects were significantly reduced, and tear production was increased by 2-D-gal both in NOD.B10.H2^b^ mice and in BALB/c mice subjected to desiccating stress (Figure 2C–F). Of note, 2-D-gal treatment rather decreased tear production, while not affecting corneal epithelial defects, in normal BALB/c mice without desiccating stress (Figure 2E,F), which suggested a differential role of α(1,2)-fucosylation at the ocular surface depending on physiologic or pathologic conditions.

### 2.3. 2-D-Gal Suppresses Inflammation in DED Mice

**Figure 3:**
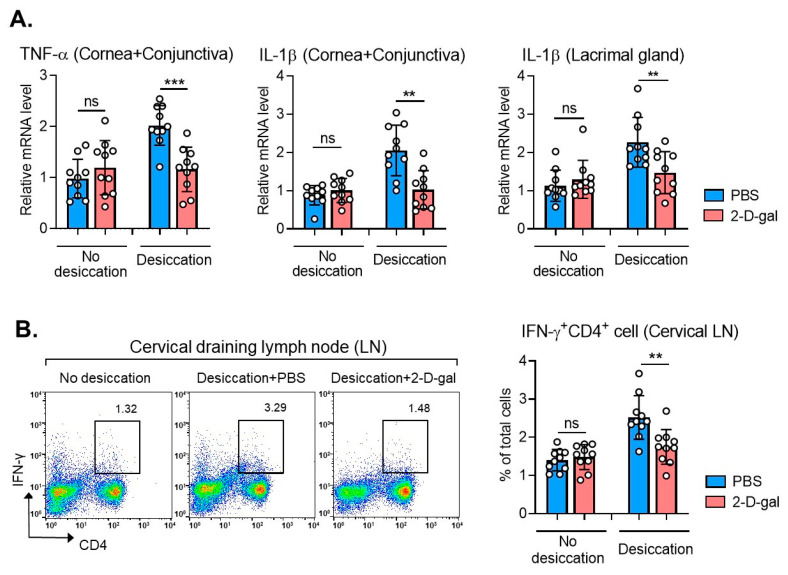
Effects of 2-D-gal on immune response at the ocular surface in DED. (**A**) Real-time RT-PCR analysis for TNF-α and IL-1β in the cornea and conjunctiva or in the lacrimal gland in BALB/c mice under physiological condition (no desiccating stress) or desiccating stress with or without 2-D-gal treatment. Shown are the relative mRNA levels to normal BALB/c mice (no desiccation, no 2-D-gal). (**B**) Representative and quantitative flow cytometry results for IFN-γ^+^CD4^+^ Th1 cells in cervical draining lymph nodes (LN). Shown are the percentages of IFN-γ^+^CD4^+^ cells out of total LN cells. A circle represents the data (mean ± SD) from an individual animal. ** *p* < 0.01, *** *p* < 0.001, ns: not significant.

Desiccating stress-induced injuries are mediated by the mucosal NF-κB activation, and subsequent production of innate inflammatory mediators leads to adaptive T cell response [24,25]. Thus, we next examined whether α(1,2)-fucosylation inhibition might affect immune response at the ocular surface in a desiccating stress-induced DED model. Real-time RT-PCR showed that 2-D-gal treatment significantly suppressed the mRNA levels of TNF-α and IL-1β in the cornea and conjunctiva and in the lacrimal gland in DED mice under desiccating stress (Figure 3A). Similar results were obtained with Th1 cells in DLNs. Flow cytometric analysis revealed that the percentages of IFN-γ^+^CD4^+^ cells in cervical DLNs were significantly reduced by 2-D-gal in DED mice (Figure 3B). However, 2-D-gal treatment did not affect the inflammatory cytokines or Th1 cells in normal mice under physiological condition (Figure 3A,B).

Collectively, the data indicate that the correction of aberrant α(1,2)-fucosylation has therapeutic effects on DED by reducing corneal epithelial defects, enhancing tear production and suppressing excessive immune response at the ocular surface.

### 2.4. Conjunctival Goblet Cell and Meibomian Gland (MG) Are Not Affected by 2-D-Gal

**Figure 4:**
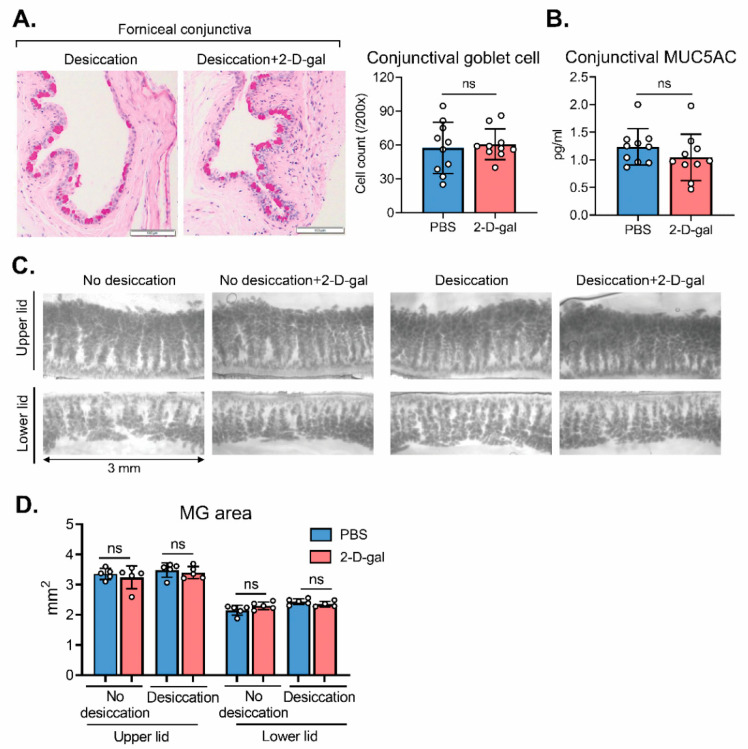
Effects of 2-D-gal on conjunctival goblet cell and meibomian gland of the eyelid. (**A**) Representative images of periodic acid–Schiff (PAS) staining of the inferior conjunctival fornix (sagittal section; scale bar: 100 μm) and quantification of PAS-stained goblet cell counts in BALB/c mice under desiccating stress with or without 2-D-gal treatment. (**B**) ELISA for conjunctival MUC5AC. (**C**) Representative transillumination meibography images. (**D**) Quantification of the meibomian gland area of the upper and lower eyelids. A circle depicts data (mean ± SD) from a single individual animal. ns: not significant.

We additionally assessed the effects of 2-D-gal on mucin-secreting conjunctival goblet cells and their production of MUC5AC. Fucosylation inhibition by 2-D-gal for 2 weeks did not change the number of conjunctival goblet cells as determined by periodic acid–Schiff (PAS) staining in DED mice induced by desiccating stress (Figure 4A). The conjunctival level of MUC5AC, a major mucin type that conjunctival goblet cells produce, was not altered by 2-D-gal as measured by ELISA (Figure 4B).

We furthermore assessed the eyelids for MGs by using transillumination meibography (Figure 4C). The areas of upper and lower MGs were not affected by 2-D-gal treatment (Figure 4C,D).

## 3. Discussion

Fucosylation is the process of transferring fucose from donor guanosine diphosphate (GDP) fucose to various acceptor molecules, including oligosaccharides, glycoproteins and glycolipids, by fucosyltransferases (FUTs) in eukaryotic organisms [26,27]. As fucosylated glycans have multiple crucial roles in various essential biological processes, an increase in fucosylation or lack thereof has been implicated in pathological processes, including blood transfusion reactions, sickle cell disease, infection, cancers and autoimmune diseases [1,2,3,4,5,6,7,8,9,10,11,12,13,28]. In this study, we found that the level of H2 antigen, one of terminally α(1,2)-fucosylated carbohydrate epitopes [29], was highly elevated at the ocular surface in DED mice and that a reduction in α(1,2)-fucosylation by 2-D-gal, a selective fucosylation inhibitor that blocks terminal α(1,2)-fucosylation [1,4,30,31], significantly ameliorated DED. These findings indicate the implication of aberrant fucosylation in DED pathogenesis and suggest aberrant fucosylation as a novel therapeutic target for DED.

One of the mechanisms underlying the therapeutic effects of 2-D-gal on DED involves the suppression of immune response at the ocular surface. In our study, the inflammatory cytokine levels in the cornea, conjunctiva and lacrimal gland, alongside Th1 cells in DLNs, were downregulated in DED mice by 2-D-gal treatment. In recent years, evidence of the involvement of fucosylation in immune cell development and function regulation has been accumulating. For example, an elegant study by Li et al. [4] revealed that terminal fucosylation was upregulated in synovial tissues from rheumatoid arthritis patients, compared to those from osteoarthritis patients, and that inhibition of terminal fucosylation by 2-D-gal caused a resolution of inflammation in collagen-induced arthritis mice. Importantly, the anti-inflammatory effects were largely mediated by inhibiting the antigen-presenting function of macrophages, leading to decreased Th17 cells in DLNs and reduced TNF-α and IL-6 levels in the serum. Since the activation of antigen-presenting cells at the ocular surface is essential for differentiation and proliferation of effector T cells in ocular DLNs in DED, it is plausible that increased α(1,2)-fucosylation in macrophages induced by desiccating stress might have contributed to immune activation at the ocular surface and subsequently aggravated DED in our model. Another possible mechanism is that aberrant fucosylation at the ocular surface in DED might have direct effects on the disruption of ocular surface epithelium, given previous studies showing that fucosylation of the intestinal epithelium is important for maintaining host–commensal symbiosis [28,32]. In both scenarios, fucosylation in the immune system and in the epithelial system plays a critical role in tissue homeostasis, reflecting that therapeutic approaches targeting fucosylation would hold promise for the development of novel therapy of ocular surface diseases including DED.

In a previous study, we characterized the ocular surface phenotype of *Fut1* KO mice that lack the gene encoding galactoside 2-alpha-L-fucosyltransferase 1, an enzyme mediating terminal fucosylation via α1,2 linkage [14]. We found that *Fut1* KO mice exhibited more severe corneal epithelial defects, increased immune response at the ocular surface and higher number of Th1 cells in DLNs, compared to WT control, in steady state and under desiccating stress. Hence, the results of our previous and current studies suggest that altered fucosylation, both upregulation and downregulation, can lead to ocular surface disruption, further emphasizing the homeostatic role of fucosylation.

2-D-gal inhibits both FUT1 and 2. Previous studies have demonstrated that FUT2 alters glycosylation patterns of the mucin MUC5AC [33] and is related to lipid metabolism [34]. In our study, 2-D-gal treatment did not change MUC5AC production or the number of conjunctival goblet cells, major producers of MUC5AC in the ocular surface. Moreover, the MG area was not altered by 2-D-gal. Thus, further research is warranted to elucidate the implication of FUT2 in ocular surface disease. Moreover, in our study, it is possible that 2-D-gal might have affected other α(1,2)-fucosylated glycans such as Lewis^b^ and Lewis^y^ antigens [35] in addition to H2 antigen, and therefore, the role of these terminal fucose-containing glycans in the ocular surface would be the subject of future research. Furthermore, it is important to investigate the role of other types of fucosylation, such as core α(1,6)-fucosylation, subterminal α(1, 3/4)-fucosylation and O-fucosylation, in ocular surface homeostasis and disease in future studies.

Fucosylation is a common process of terminal glycan modification responsible for cellular responses to environmental stimuli as well as cellular growth and differentiation. Alterations in fucosylated glycan composition are frequently linked to many diseases, including DED. A better understanding of the physiologic and pathologic roles of fucosylation at the ocular surface would help identify novel biomarkers and therapeutics for ocular surface disorders.

## 4. Materials and Methods

### 4.1. Animal Model

The animal experiment protocols were approved by the Institutional Animal Care and Use Committee of Seoul National University Biomedical Research Institute (IACUC No. 19-0240-S1A1; Approval date: 1 April 2020). Animal experiments were performed in accordance with the Association for Research in Vision and Ophthalmology (ARVO) Statement for Use of Animals in Ophthalmic Vision and Research.

As an ocular SjS model, 12-week-old male NOD.B10.H2^b^ mice (Jackson Laboratories, Bar Harbor, ME, USA) were used because the strain spontaneously exhibits dacryoadenitis and aqueous-deficient DED [36]. For controls, 12-week-old male C57BL/6 mice (Koatech, Seoul, Korea) were used.

For induction of a desiccating stress-related DED model, 28-week-old male BALB/c mice (Koatech) were housed in a dry cage equipped with perforated plastic screens and a dehumidifier. The airflow from an electric fan entered the cage through the screens for 24 h, and the humidity inside the cage was maintained at 30 to 35%. Additionally, an IP injection of scopolamine hydrobromide (0.5 mg/0.2 mL, Sigma-Aldrich, St. Louis, MO, USA) was performed three times a day.

For inhibition of fucosylation, 2-D-gal (200 μL, 250 mg/kg body weight, Sigma-Aldrich), which blocks terminal α(1,2)-fucosylation [1,4,25,26], was injected IP into mice every other day for 2 weeks (a total of 7 injections per mouse). The same volume of phosphate-buffered saline was injected in the control group in the same manner.

### 4.2. Clinical Examination of Corneal Epithelial Defects

The ocular surface was observed for corneal epithelial defects under an operating microscope and photographed. For quantification of corneal epithelial defects, a drop of 3% Lissamine Green B dye (Sigma-Aldrich) was applied to the corneal surface, and the stained epithelial defects were graded in a blinded manner according to the standardized scoring system (score 0: no staining, score 0.5: trace, score 1: less than one-third, score 2: less than two-thirds, and score 3: more than two-thirds staining of the cornea) [37].

### 4.3. Measurement of Tear Production

The amount of tear secretion was quantified by the phenol red thread test, where a phenol red-impregnated cotton thread (FCI Ophthalmics, Pembroke, MA, USA) was inserted into the lateral canthus of an eye for 60 s, and the length of cotton thread wetted by tears was measured in millimeters.

### 4.4. Transillumination Meibography

The morphology of the MG was observed and the MG area was calculated by transillumination meibography as previously described [38]. Briefly, both upper and lower eyelids were gently excised, and the skin was removed as much as possible. The eyelids were placed between two microscopic slides over a wide-spectrum white light source (LED plate). Light transmitted through the eyelids was captured using a microscope equipped with an infrared filter (Hoya R72, TOKINA Co. Ltd., Tokyo, Japan) and a charge-coupled device (CCD) camera (acA1600-20 um, Basler Inc., Ahrensburg, Germany). The area of interest (the central 3 mm area) was analyzed using ImageJ software (Version 1.52a; NIH Image, Bethesda, MD, USA). The MG area was semi-automatically selected using the Wand tool (tolerance range of 5 to 30) of the software.

### 4.5. Western Blotting

For protein extraction, the tissues were sonicated on ice in RIPA buffer (Biosesang, Seongnam, Korea) supplemented with Halt Protease Inhibitor Cocktail (Thermo Fisher Scientific, Waltham, MA, USA). After centrifugation at 12,000 rpm at 4 °C for 20 min, cell lysates were prepared and analyzed for protein concentration by Bradford assay. A total of 40 μg protein was fractionated by SDS-PAGE on 8–16% Tris–glycine gel (Komabiotech, Seoul, Korea), transferred to PVDF membrane (Thermo Fisher Scientific) and blotted with antibodies against H2 antigen (1:500, Cat No. 78438, Santa Cruz Biotechnology, Santa Cruz, CA, USA) or β actin (1:1000, Santa Cruz Biotechnology) [14].

### 4.6. Enzyme-Linked Immunosorbent Assay (ELISA)

The level of MUC5AC in the conjunctiva was measured by ELISA. The protein was extracted from the conjunctiva as described above and assayed for the concentration of MUC5AC by using mouse MUC5AC ELISA kit (Novus Biologicals, Centennial, CO, USA).

### 4.7. Real-Time Reverse Transcription Polymerase Chain Reaction (RT-PCR)

For RNA extraction, the tissues were cut into small pieces with microscissors and lysed in RNA isolation reagent (RNA-Bee, Tel-Test Inc., Friendswood, TX, USA). Then, the tissues were homogenized with an ultrasound sonicator (Ultrasonic Processor, Cole Parmer Instruments, Vernon Hills, IL, USA). Total RNA was extracted with an RNeasy Mini kit (Qiagen, Valencia, CA, USA) and was converted to the first-strand cDNA by reverse transcription using High Capacity RNA-to-cDNA kit (Applied Biosystems, Carlsbad, CA, USA). The cDNA was analyzed by real-time PCR amplification for TNF-α and IL-1β on an ABI 7500 Real Time PCR System (Applied Biosystems). All probe sets were purchased from Applied Biosystems (TaqMan Gene Expression Assay kits). Mouse-specific GAPDH was used as internal controls.

### 4.8. Flow Cytometry

To obtain single-cell suspension, the bilateral cervical draining lymph nodes (DLNs) were minced between the frosted ends of two glass slides in the media containing RPMI-1640 (WelGENE, Daegu, Korea) and 10% fetal bovine serum (Gibco, Grand Island, NY, USA) and were then filtered through a cell strainer. The resultant single-cell suspensions were stained with fluorescence-conjugated antibodies against CD4-PE cy7 eBioscience, Waltham, MA) and IFN-γ-FITC (eBioscience). For intracellular IFN-γ staining, the cells were prestimulated for 5 h at 37 °C with 5 μg/mL anti-CD3 (BD Pharmingen, San Diego, CA) and 5 μg/mL anti-CD28 (BD Pharmingen) in the presence of Cell Stimulation Cocktail including phorbol 12-myristate 13-acetate (PMA) and ionomycin (Cat No. 00-4970, eBioscience). The stained cells were assayed by a flow cytometer (S1000EXi Flow Cytometer, Stratedigm, San Jose, CA, USA) and were analyzed using FlowJo.

### 4.9. Histology

The whole eyeball including the superior and inferior forniceal conjunctiva was excised and fixed in 10% formaldehyde. The tissues were sliced into 4 mm thick sections through superior and inferior conjunctival fornices and were subjected to PAS staining (Abcam, Cambridge, MA, USA). The number of PAS-stained goblet cells was counted in 4 different sections of the same eye, and the average number per section in each eye was determined as the goblet cell count [39].

### 4.10. Statistical Analysis

Prism software v.9.0.0 (GraphPad, La Jolla, CA, USA) was used for statistical tests. To compare the means of two groups, data were analyzed by the two-tailed unpaired t-test. The data are presented as mean ± SD, and *p* values < 0.05 were considered statistically significant.

## Data Availability

All data is contained within the article. Raw or additional data will be provided upon request.

## Abbreviations

2-D-gal	2-Deoxy-D-galactose
CCD	Charge-coupled device
DED	Dry eye disease
FUT	Fucosyltransferase
H2 antigen	Type 2 H antigen
IP	Intraperitoneal
MG	Meibomian gland
PAS	Periodic acid–Schiff
SjS	Sjögren’s syndrome
WT	Wild-type
